# A randomised trial of the influence of racial stereotype bias on examiners’ scores, feedback and recollections in undergraduate clinical exams

**DOI:** 10.1186/s12916-017-0943-0

**Published:** 2017-10-25

**Authors:** Peter Yeates, Katherine Woolf, Emyr Benbow, Ben Davies, Mairhead Boohan, Kevin Eva

**Affiliations:** 1 Medical Education Research, School of Medicine, David Weatherall Building, Keele University, Newcastle under Lyme, ST5 5BG UK; 2 Acute and Respiratory Medicine at Pennine Acute Hospitals NHS Trust, Bury, UK; 3 University College London Medical School, University College London, London, UK; 4 Division of Medical Education, University of Manchester, Manchester, UK; 5 Central Manchester University Hospitals NHS Foundation Trust, Manchester, UK; 6 North Devon Healthcare NHS Trust, Barnstaple, UK; 7 School of Medicine, Dentistry and Biomedical Sciences, Queens University Belfast, Belfast, UK; 8 Centre for Health Education Scholarship, Faculty of Health, University of British Columbia, Vancouver, Canada

**Keywords:** Medical education, Assessment, Differential attainment, Ethnicity, Stereotypes

## Abstract

**Background:**

Asian medical students and doctors receive lower scores on average than their white counterparts in examinations in the UK and internationally (a phenomenon known as “differential attainment”). This could be due to examiner bias or to social, psychological or cultural influences on learning or performance. We investigated whether students’ scores or feedback show influence of ethnicity-related bias; whether examiners unconsciously bring to mind (activate) stereotypes when judging Asian students’ performance; whether activation depends on the stereotypicality of students’ performances; and whether stereotypes influence examiner memories of performances.

**Methods:**

This is a randomised, double-blinded, controlled, Internet-based trial. We created near-identical videos of medical student performances on a simulated Objective Structured Clinical Exam using British Asian and white British actors. Examiners were randomly assigned to watch performances from white and Asian students that were either consistent or inconsistent with a previously described stereotype of Asian students’ performance. We compared the two examiner groups in terms of the following: the scores and feedback they gave white and Asian students; how much the Asian stereotype was activated in their minds (response times to Asian-stereotypical vs neutral words in a lexical decision task); and whether the stereotype influenced memories of student performances (recognition rates for real vs invented stereotype-consistent vs stereotype-inconsistent phrases from one of the videos).

**Results:**

Examiners responded to Asian-stereotypical words (716 ms, 95% confidence interval (CI) 702–731 ms) faster than neutral words (769 ms, 95% CI 753–786 ms, *p* < 0.001), suggesting Asian stereotypes were activated (or at least active) in examiners’ minds. This occurred regardless of whether examiners observed stereotype-consistent or stereotype-inconsistent performances. Despite this stereotype activation, student ethnicity had no influence on examiners’ scores; on the feedback examiners gave; or on examiners’ memories for one performance.

**Conclusions:**

Examiner bias does not appear to explain the differential attainment of Asian students in UK medical schools. Efforts to ensure equality should focus on social, psychological and cultural factors that may disadvantage learning or performance in Asian and other minority ethnic students.

**Electronic supplementary material:**

The online version of this article (doi:10.1186/s12916-017-0943-0) contains supplementary material, which is available to authorized users.

## Background

Medical students and doctors from black and minority ethnic (BME) backgrounds, including those from Asian groups, perform less well on average than their white counterparts in assessments [1]. These ethnic differences are found among British-trained students, not just in international medical graduates, and reflect similar findings from the Netherlands [2], the USA [3] and Australia [4]. This effect is small but consistent [5]; occurs in both written and performance-based assessments [6, 7]; at different stages of the educational continuum [8, 9]; and is incompletely explained by prior attainment [8]. The reasons for this differential attainment are unclear but broadly could arise either because exam systems are biased against BME students or because social, psychological or cultural factors result in a lower average standard of performance by BME trainees. Understanding which effect is responsible is vital to successfully targeting interventions to ensure equality.

Research in other domains has repeatedly demonstrated humans’ susceptibility to unconscious bias when making judgements about individuals from negatively stereotyped groups [10]. Woolf et al. [11] have described some medical educators in the UK holding stereotyped views of the performance of BME medical students, in which Asian students are conceived as often having good factual knowledge but poor communication skills. This is pertinent; stereotypes are readily activated when making judgements [12] and can bias the information a person attends to [13], the judgement they reach [14] and their memory of what occurred [15], with such memory bias serving to perpetuate stereotypes [16]. Stereotypes often influence judgements beyond conscious awareness [17] and are more likely to have an influence when judgements are mentally taxing [18], as is the case for examiners during medical exams [19]. If any such bias influences medical student exams, as well as influencing the scores that BME students receive, it could also result in provision of feedback that is more negative than white students receive. Prior research on assessment in medical education has shown that other judgemental biases (contrast effects) influence the strength of language used in feedback in a similar pattern to influences on scores [20]). As a result, if a stereotype bias occurs, we may expect to see its influence on the valence of feedback as well as on scores. Equally, due to recollection bias, feedback could focus on stereotyped aspects of performance, thereby distorting the conveyed message and creating a potential determinant of students’ self-efficacy and future learning strategies/performances. It is entirely plausible, therefore, that differential attainment could arise due to examiner bias derived from stereotyping of BME students.

Conversely, a number of retrospective analyses of exam data have gone some way to refuting an influence of examiner bias: Woolf et al. [6] found similar degrees of differential attainment by BME students on both machine-marked written exams and examiner-based performance exams; McManus et al. [21] found that only 3/1790 examiners showed evidence of ethnic bias compared to other examiners observing the same candidates; Denney et al. [22] found that very few examiners appeared to favour candidates of their own sex or ethnicity. No prior studies have examined the potential of examiner bias under conditions of experimental control. In this study we sought to determine whether examiners show evidence of stereotype activation when examining BME students; whether stereotype activation is dependent on the student’s behaviour matching the described stereotype; whether examiners’ scores or feedback show any evidence of ethnicity bias; or whether examiners’ memory of performances suggests any influence of stereotypes on their judgements.

### Research questions

1. Do examiners activate stereotypes relating to students’ ethnicity whilst judging students’ performances?
2. Is any such stereotype activation dependent on students’ performances matching described stereotypes?
3. Are the (a) scores, (b) valence of feedback, or (c) focus of feedback that examiners give to students’ performances influenced by students’ ethnicity?
4. Do examiners’ memories of students’ performances show evidence of stereotype bias?

To operationalise these questions, we chose to focus on “British Asian” students, defining this term as individuals with recent heritage from the Indian subcontinent who had been born or educated within the UK. We chose this group because they are the largest group of BME students in UK medical schools and because of the existence of academic literature describing a stereotype of this group within medical education [11].

## Methods

### Study design

We used a two-group, double-blinded, randomised Internet-based experimental design.

### Participants, recruitment and consent

Participants were current UK undergraduate Objective Structured Clinical Exam (OSCE) examiners. Inclusion criteria were as follows: being a licensed doctor within the UK; having previously received training as an OSCE examiner; having examined a summative OSCE at a UK medical school within the last 2 years; being comfortable to assess both communication skills and knowledge. Recruitment was undertaken by email; medical schools around the UK disseminated the invitation to OSCE examiners. Interested individuals registered on the study website and received the Participants Information Sheet. Consent was obtained online via the study website prior to participation. Participants were offered a £20 shopping voucher for study completion. As prior knowledge of the study’s premise could have biased examiners’ responses, participants were blinded to the study intervention by the use of a deceptive premise that simply stated: “we’re interested in understanding more about how OSCE examiners make judgements on clinical performance when they are assessing OSCEs”, with an assurance that a fuller explanation would follow.

### Measures, procedure and hypotheses

An overview of the study design and procedure is shown in Table 1. Details of the validation of the stimulus materials and measures are given in Additional file 1.

**Table 1:** Overview of study design

	Instructions	Performances^a^			Lexical decision task	Recollection	Demographics	Debrief
Group		K+/C–	K–/C+	Mixed		Mixed		
Stereotype-consistent		Asian_1_	White_1_	Asian_2_		Asian		
Stereotype-inconsistent		White_1_	Asian_1_	White_2_		White		

^a^Order of performance was counterbalanced within groups (half of each group saw K+/C–, K–/C+, mixed; the other half saw mixed, K–/C+, K+/C–)

*K+* good knowledge demonstrated in performance, *K–* poor knowledge demonstrated

*C+* good communication demonstrated in performance, *C–* poor communication demonstrated

#### Scripted videos of OSCE performances of white and Asian medical students

We created videos of scripted medical student performances on a simulated OSCE station, in which a young woman attends her general practitioner to discuss a new diagnosis of type 1 diabetes mellitus. The scenario required the student to both demonstrate accurate knowledge of type 1 diabetes and to display empathy and good communication skills. The scenario is described in Additional file 1: Section 1.

Three separate scripts were created by a clinical educator (PY): one showed good factual knowledge and poor communication skills (K+/C–) (i.e. a performance consistent with a described stereotype of Asian students’ performance [11]); one showed poor factual knowledge and good communication skills (K–/C+) (i.e. a performance inconsistent with a described stereotype of Asian students’ performance); and one showed a mixture of both good and poor knowledge and communication (mixed). The scripting was done by drawing from PY’s clinical knowledge and experience of assessing medical trainees at different stages of training and aimed to represent a plausible, authentic performance in an OSCE by an undergraduate medical student. Scripts were reviewed by a panel of six experienced clinical educators who scored the knowledge and communication that was displayed in each script. Details of their scores can be seen in Additional file 1: Section 2.

All scripts were performed and filmed twice: once by an actor who was white with a white British accent, and once by an actor who was Asian with a British Asian accent, giving a total of six performance videos. Four separate actors were involved: two men and two women. Both men performed in both the K+/C– and K–/C+ videos, but within groups participants saw one man for the first performance and the other man for the second performance. The women performed the two versions of the mixed performance. As a result, participants saw a different actor in each video. The similarity of the British Asian and white versions of each performance were judged by a panel of eight experienced clinical educators. All paired performances were judged to be at least “highly similar”. Details of this validation exercise can be seen in Additional file 1: Section 2.

Examiners were randomised to two groups by the study Internet site, using a random number generator with a variable maximum between-group discrepancy function. A stereotype-consistent group (Group A) saw Performance 1 (K+/C–) with an Asian student and Performance 2 (K–/C+) with a white student. A stereotype-inconsistent group (Group B) saw Performance 1 with a white student and Performance 2 with an Asian student. In order to test the hypothesis relating to memory, both groups also saw the mixed performance, featuring an Asian student in Group A and a white student in Group B. To prevent order effects, the order in which performances were presented within groups was balanced, with equal numbers seeing them in order 1: K+/C–, K–/C+, mixed; and order 2: mixed, K–/C+, K+/C–.

#### Performance scoring

Prior to seeing the videos, examiners were provided with briefing material describing the OSCE scenario, desirable student behaviours, key case-related information and the mark sheet. This material is available in Additional file 1: Section 1.

Examiners watched the three performances they were assigned online. After each performance, examiners scored the observed student on four domains (two relating to communication skills and two relating to factual knowledge) on 7-point rating scales end-anchored with “no elements done” and “all elements done well”. The two domains were collapsed to give average ratings of communication and knowledge for each participant for each performance. Examiners also provided an overall global rating on a 7-point scale anchored with Fail (1, 2), Borderline (3), Pass (4), Good (5), Excellent (6, 7). Finally, examiners were asked to “provide up to three suggestions for improvement” as free text feedback. The scoring format was based on the standard format of OSCE mark sheets from one medical school which recruited participants, and will have been very familiar to these examiners. Whilst it may have been less familiar to other participants, it was similar to typical domain-based mark sheets.

We hypothesised that there would be a main effect of student ethnicity, with Asian students receiving lower scores than white students in both groups (Hypothesis 1).

#### Free text feedback

Free text feedback comments were segmented and analysed by content analysis. Each portion of feedback was segmented by a single researcher into pieces of feedback that were judged to contain a single concept. Each feedback segment was uniquely labelled, and then two researchers independently coded each segment for its focus (communication, factual-knowledge or general) and valence (positive, negative or neutral). Both researchers met repeatedly to discuss and develop a shared interpretation of the data. All analysis was done blind to study group and the ethnicity of the student to whom the feedback had been given. Agreement between the researchers was calculated using Cohen’s kappa. Remaining discrepancies were resolved through discussion prior to unblinding. Once the analysis was complete, the balance of focus was calculated for each student performance for each participant, by allocating a score of +1 to communication-focused segments, –1 to knowledge-focused segments and 0 to general segments, and then summing the segment scores. As a result, feedback with a positive score focused more on communication than knowledge and feedback with a negative score focused more on knowledge than communication. The same procedure was used for the valence of feedback by allocating positive segments +1, negative segments –1 and neutral segments 0. This resulted in a focus and valence score for the feedback given to each student performance by each participant.

On the basis that judgemental bias tends to have a correspondingly positive or negative influence on feedback, we hypothesised that the valence of feedback to Asian students would be comparatively negative compared to the valence of feedback to white students (Hypothesis 2a).

On the basis that examiners tend to focus feedback on areas of weak performance, we hypothesised that the focus of feedback to Asian students would incline more towards communication skills than the focus of feedback for white students (Hypothesis 2b).

#### Test of stereotype activation

After scoring three performances, examiners performed a lexical decision task to gain a measure of their mental activation of Asian stereotypes (or, put more simply, whether they had brought to mind a stereotype of “Asian-ness” whilst judging students’ performances). Lexical decision tasks are a well-established measure of stereotype activation within psychological research. Numerous previous studies have shown that when a stereotype is activated (for example by someone coming into contact with a person from a stereotyped group), concepts associated with that stereotype become more readily available in the mind of the person who experiences the stereotypical thoughts. As a result they tend to respond to stereotype-related concepts more quickly than neutral concepts [23]. Lexical decision tasks rely on the premise that when a stereotype has been mentally activated, people can respond more quickly to words associated with the stereotype than to neutral words. This enables detection of stereotype activation. The task consisted of 45-letter strings of which 30 were words and 15 were non-words. Of the 30 words, 15 were “stereotype words” (words associated with stereotypes of south-Asian people in the UK) and 15 were “neutral” (words that were unrelated to Asian stereotypes). All strings were presented in the same random order to both groups. These words, along with evidence supporting the validity of their “neutral” or “Asian” association, can be viewed in Additional file 1: Section 2.

After one practice trial, examiners were asked to determine whether presented strings of letters were either a real word or place name in the English language, or a “non-word” (a string of letters with no meaning), by pressing “D” or “K” on the keyboard, respectively. Examiners were asked to work as quickly but as accurately as possible. To prevent demand characteristics, this was presented as a “test of concentration”; examiners were not made aware that some words were related to an Asian stereotype. All responses were timed locally, using the clock within the participant’s computer, thus negating effects of Internet bandwidth.

We hypothesised that participants would have faster response times to stereotypical words than neutral words (Hypothesis 3a).

We hypothesised that Group A (who had seen comparatively stereotype-consistent performances) would have faster response times to stereotypical words than Group B (who had seen comparatively stereotype-inconsistent performances) (Hypothesis 3b).

#### Test of memory

Following the stereotype activation task (approximately 5 minutes), examiners completed a recognition-based memory test. Examiners were asked to read 40 quotes ostensibly from the “mixed” performance video, and indicate whether they had occurred in the performance or were invented, by marking them as “true” or “false”. Of the presented statements 20 were accurate quotes from the mixed performance (real) and 20 did not appear in any of the three videos (invented). Group A had seen the mixed performance played by an Asian female student, whilst Group B had seen the performance played by a white female student. For both the real and invented statements, half were consistent with the literature-based stereotype of Asian students’ performance (a balanced mixture of accurate factual knowledge and examples of poor communication), and half were inconsistent with the stereotype of Asian students’ performance (a balanced mix of inaccurate factual knowledge and examples of good communication). Evidence supporting the validity of these constructs is presented in Additional file 1: Section 2. The manipulated video presentation order meant that within each group exactly half of examiners saw the mixed video first and half saw the mixed video last, thereby balancing any effect of video order on memory across groups.

When stereotypes influence memory, they cause two opposite effects: statements which are real, but *inconsistent* with the stereotype, seem unexpected, making them more salient and increasing their rate of recognition; conversely, statements that are invented, but *consistent* with the stereotype, seem plausible, also increasing their rate of recognition [15]. Consistent with this, we compared (1) the proportion of real, stereotype-inconsistent responses marked “True” and (2) the proportion of invented, stereotype-consistent responses marked “True” between groups that had seen an Asian vs a white student for the mixed performance.

We hypothesised that examiners in Group A (stereotype-consistent group) would mark a higher proportion of real stereotype-inconsistent statements and invented stereotype-consistent statements as “True” than examiners in Group B (stereotype-inconsistent group) (Hypothesis 4).

#### Demographics and debrief

After completing all tasks, examiners provided demographic data including their own ethnicity using UK Office for National Statistics ethnicity categories. Participants were asked to indicate in free text what they thought the study was testing, before being provided a description of the study’s premise. Repeat consent was then sought.

### Analysis

#### Performance scores

We analysed scores using generalised linear modelling with generalised estimating equations (GLM GEE), with within-subject variables of performance (K+/C–, K–/C+, mixed) and student ethnicity. Co-variate analyses based on demographic data were performed to exclude confounding. These analyses were performed on overall scores, communication scores and knowledge scores, respectively. The influence of students’ ethnicity on the focus and valence of examiners’ feedback was then compared sequentially using GLM GEE, in a similar analysis to that used for scores, but with ‘focus’ and then ‘valence’ as the dependent variables.

As no prior data were available for power calculations, the study was powered based on interim examination of the groups’ standard deviations (without use of inferential tests) to determine how large a sample would be required to find a statistically significant difference of 0.35 out of 7.0 in scores on the “overall scores” measure. This difference would be similar to the difference in scores observed between BME students and white students in a communication-focused OSCE exam by Wass et al. [7] with a similar effect size to that seen in the meta-analysis by Woolf et al. [5].

#### Stereotype activation

We used repeated-measures analysis of variance (ANOVA) to compare participants’ mean response times for stereotype words and neutral words (within-subject variable) between groups (between-subject variable). Using the procedure described by Mussweiler and Epstude [24], responses to individual target words were excluded if they were either incorrectly identified (for example if a participant indicated a non-word when the target was a word) or were greater than 2 standard deviations (SD) from the mean response time for that category of word (interpreted as erroneous responses or distraction). Median exclusion rates were compared between groups using the Mann-Whitney U test.

#### Recollection

We used two independent-group univariate ANOVAs to compare (1) the proportion of *real*, stereotype-*inconsistent* statements marked “True” by each group and (2) the proportion of *invented*, stereotype-*consistent* statements marked “True” by each group.

## Results

### Participants

Participants were recruited between November 2014 and June 2015, and recruitment closed when the recruitment target was achieved. A total of 181 examiners enrolled, and 159 completed the study. Responses by all participants who completed the study were included in all analyses. Completing participants came from 20 of the UK’s 33 medical schools and from a broad range of clinical specialities. The majority of examiners were recruited from 4 medical schools (a total of 93 out of 169). These are denoted A–D in Table 2. The remaining 16 schools contributed 7 or fewer participants each. Groups were equal in size (Group A: 92 enrolled, 12 dropped out, 80 completed vs Group B: 89 enrolled, 10 dropped out, 79 completed). As enrolled participants could leave the website without giving reasons, no explanations were obtained for dropouts. The study groups were similar in all measured demographics: year of qualification, years of OSCE examining experience and frequency of OSCE examining per year (see Table 2). Participants were predominantly of white ethnicity but also included individuals from a range of other ethnicities. To facilitate baseline comparisons, ethnicities were grouped as “white”, “Indian subcontinent” and “other minority ethnic individuals”. Numbers of participants in each of these categories did not vary between groups. These data are also presented in Table 2. Examination of participants’ responses in the debrief phase indicated that only two participants (1.2% of all completed respondents) guessed the study’s true purpose; therefore, no participants were excluded, given that all consented to ongoing inclusion of their data.

**Table 2:** Comparison of participant characteristics between groups

Characteristic	Group A (viewed stereotype-consistent performances)	Group B (viewed stereotype-inconsistent performances)	Significance
Sex:	Frequency		*p* (chi sq.)
Male	40 (51%)	29 (37%)	0.078
Female	39 (49%)	50 (63%)	
Clinical speciality:			
Anaesthetics	7	2	0.09 (Fisher exact)
Diagnostic specialities	5	2	
Hospital medicine	24	31	
Surgery	10	4	
Emergency medicine	3	0	
Child health	3	7	
Women’s health	2	7	
General practice	15	17	
Psychiatry	4	3	
Non-hospital medical specialities	1	0	
Public health	0	1	
Other	6	5	
Medical school:			
A	5	13	0.15
B	20	21	
C	9	4	
D	10	12	
Others	34	26	
Participant ethnicity:			
White	61	66	0.11 (Fisher exact)
Indian subcontinent	11	7	
Other minority ethnicity	6	1	
Prefer not to say	2	5	
	Median		*p* (Mann-Whitney U)
Year of qualification	1995	1992	0.75
Years examining OSCEs	5	5	0.47
OSCEs/year	2	2	0.79

### Evidence that examiners activate mental stereotypes of students

As it is pertinent to the consideration of further results, we will present these data first. Participants’ responses to 8.2% of target words were excluded due to being incorrect or erroneous (> ± 2 SD from category mean); median exclusion rates showed no significant difference between groups (*p* = 0.147). Examiners’ response times to stereotype words (mean = 716 ms (95% CI 702–731 ms)) were faster than their response times to neutral words (769 ms (753–786 ms), *F* = 220.4, *p* < 0.001). No difference was observed, however, between groups in their response times: Group A, 750 ms (729–772) vs Group B, 735 ms (714–756 ms), *p* = 0.32, and the interaction of word type x group was also non-significant (*p* = 0.55). The same pattern or results were observed for both white and non-white participants. As a result, Hypothesis 3a was supported and 3b was refuted: examiners in both groups showed evidence of stereotype activation, regardless of whether the performances they had seen by Asian students had been stereotype-consistent or stereotype-inconsistent.

### Scores

Score data consisted of 18 separate distributions: 3 performances x 3 measures (knowledge; communication; overall) x 2 groups. Domain scores followed the scripted discordant patterns: K+/C– knowledge mean = 5.6 (95% CI 5.5–5.8) and communication = 2.5 (2.3–2.6); K–/C+ knowledge mean = 3.0 (2.9–3.2) and communication mean = 5.7 (5.6– 5.8); mixed knowledge mean = 3.1 (2.9–3.2) and communication mean = 3.6 (3.4–3.7). Knowledge scores (*p* < 0.001) and communication scores (*p* < 0.001) differed statistically significantly between performances. These data are shown in Table 3.

**Table 3:** Comparison of scores (knowledge, communication, overall scores) and feedback (focus and valence) by performance and group

Performance	Group A (viewed stereotype-consistent performances)	Group B (viewed stereotype-inconsistent performances)
Performance 1: K+/C–	Asian student	White student
Scores		
Knowledge	5.6 (5.3–5.9)	5.6 (5.4–5.9)
Communication	2.4 (2.2–2.6)	2.5 (2.3–2.7)
Overall	3.2 (3.0–3.5)	3.1 (2.8–3.4)
Feedback		
Focus	2.7 (2.5–3.0)	2.7 (2.4–2.9)
Valence	–2.6 (–2.9 to –2.4)	–2.6 (–2.9 to –2.4)
Performance 2: K–/C+	White student	Asian student
Scores		
Knowledge	3.0 (2.8–3.3)	3.0 (2.8–3.2)
Communication	5.6 (5.5–5.8)	5.7 (5.6–5.9)
Overall	3.4 (3.1–3.6)	3.3 (3.1–3.5)
Feedback		
Focus	–0.3 (–0.5 to 0.0)	–0.4 (–0.7 to 0.0)
Valence	–1.7 (–2.0 to –1.4)	–1.8 (–2.1 to –1.5)
Performance 3: mixed	Asian student	White student
Scores		
Knowledge	3.1 (2.9–3.3)	3.1 (2.9–3.3)
Communication	3.5 (3.3–3.7)	3.6 (3.4–3.8)
Overall	2.9 (2.7–3.0)	2.8 (2.6–3.0)
Feedback		
Focus	1.6 (1.3–1.9)	1.5 (1.2–1.9)
Valence	–2.8 (–3.1 to –2.6)	–3.0 (–3.3 to –2.7)

All comparisons are non-significant

### Influence of students’ ethnicity on examiners’ scores

Comparison of performance scores showed no difference due to student ethnicity. The average knowledge score when performances were acted by Asian students was 3.9 (95% CI 3.8–4.0) vs 3.9 (3.8–4.0) when acted by white students (*p* = 0.77). The average communication score when the performances were acted by Asian students was 3.9 (3.8–4.1) vs 3.9 (3.7–4.0) when acted by white students (*p* = 0.31). The average overall scores when the performances were acted by Asian students was 3.1 (2.9–3.3) vs 3.1 (3.0–3.3) when acted by white students (*p* = 0.88). The scores for each measure on each performance by each group are shown in Table 3. Statistical examination for potential confounding effects of examiners’ sex or ethnicity showed that neither of these variables confounded the comparisons of interest. The study had 81% power to detect a difference of 0.35 out of 7.0 on the assessment scale, equivalent to an effect size of *d* = 0.32. As a result, Hypothesis 1 was not supported, suggesting that the Asian stereotypes, which the lexical decision task suggested were activated among examiners, did not influence their scoring.

### Influence of students’ ethnicity on examiners’ feedback

Agreement between the two analysts regarding how to categorise feedback statements was high (Cohen’s kappa for ‘Focus’ codings = 0.85; quadratic weighted Cohen’s kappa for ‘Valence’ codings = 0.92). The focus of feedback varied by performance, indicating that examiners generally focused their feedback on weaker areas of performance (see Table 3). Students’ ethnicity had no influence on the focus of feedback, with both groups receiving more feedback comments on communication than on factual knowledge: Asian students, 1.3 (1.2–1.5) vs white students, 1.3 (1.1–1.5), *p* = 0.87. The interaction of performance by student ethnicity for feedback focus was also non-significant, *p* = 0.82. All performances received more negative than positive feedback. There was no influence of students’ ethnicity on the valence of feedback: Asian students –2.4 (–2.6 to –2.3); white students –2.4 (–2.6 to –2.3), *p* = 0.82. The interaction of performance by student ethnicity was also non-significant for feedback valence at *p* = 0.57. As a result, neither Hypotheses 2a or 2b were supported, suggesting that the Asian stereotypes which examiners activated did not influence their provision of feedback.

### Influence of students’ ethnicity on examiners’ memories for the mixed performance

Participants agreed with real statements more frequently than with invented statements (real 72.5% (70.6–74.4%); invented 32.7% (30.8–34.5%), *F* = 871.3, *p* < 0.001). Statements that are real but stereotype *inconsistent* are theoretically expected to be more memorable if a stereotype has influenced memory, as they seem unexpected, and so achieve increased saliency; no between-group difference occurred in recognition rates of such statements: Group A (recalling an Asian student on the mixed performance) 75% (72–79%); Group B (recalling a white student on the mixed performance) 78% (75–82%), *F* = 1.63, *p* = 0.20. Invented statements that are stereotype-consistent are theoretically expected to be endorsed more often if stereotypes influence judgements because they seem particularly plausible. No between-group difference occurred in these statements: Group A (recalling an Asian student on the mixed performance) 29% (24–33%); Group B (recalling a white student on the mixed performance) 32% (27–36%), *F* = 0.84, *p* = 0.36. Recognition data are shown in Table 4. As a result, Hypothesis 4 was not supported, suggesting that the activated Asian stereotypes examiners demonstrated via the lexical decision task did not influence their memories of performances.

**Table 4:** Proportions of each statement type marked as true, by group, within the recognition test of memory

	Proportion of statements marked “True” (95% CIs)	
Statement type:	Group A (recalling Asian student)	Group B (recalling white student)
Real statements		
Stereotype-consistent	69% (65–73%)	68% (64–71%)
**Stereotype-inconsistent**	**75% (71**–**79%)**	**78% (75**–**81%)**
Invented statements		
**Stereotype-consistent**	**29% (24–33%)**	**32% (27–36%)**
Stereotype-inconsistent	34% (31–36%)	37% (34–40%)

Theorised comparisons are highlighted in boldface and are non-significant

## Discussion

### Summary of results

For the first time in a double-blinded, randomised, controlled study, we have compared the influence of students’ ethnicity (white vs British Asian) on (1) the scores and feedback that OSCE examiners give to simulated undergraduate student OSCE performances and (2) examiners’ cognitive processing of those performances including their recollection accuracy and activation of an Asian stereotype when examining Asian students. Examiners showed evidence of stereotype activation (either reflecting a generalised activation or activation induced by exposure to the Asian students in our stimuli), regardless of whether Asian students’ performances were consistent with a described stereotype [11]. Despite this, we found no effect of students’ ethnicity on the scores that were assigned; the valence or focus of the feedback that students received; or any evidence of bias in the recollections of performances by examiners. Our findings are partly consistent with both the extensive literature in social psychology, which describes the prevalence of stereotyping in judgements, and the literature in medical education, which has suggested that examiner bias is not responsible for differential attainment by BME students. The fact that this study and previous uncontrolled observational field studies [5, 9, 22] have found consistent results by different methods helps to support this conclusion.

### Practical and theoretical implications

A recent prominent legal case in the UK (see [25]) has reaffirmed an important principle of equality law: that the absence of direct discrimination in an exam system does not mean educational organisations are absolved of responsibility for differential attainment. Instead, the education system is responsible to ensure equality of opportunity for minority groups by addressing indirect discrimination and providing reasonable support. The average underperformance of BME students and training-grade doctors in UK medical exams is a robust observation that should not be ignored. Whilst a single study cannot definitively exclude the possibility of examiner bias in all circumstances, this study tends to suggest that efforts to address differential attainment should focus on factors which either reduce learning opportunities or hinder performance for BME students. A variety of potential avenues could be explored. For example, Vaughan et al. [26] showed that the social networks of BME students in UK medical schools may produce a relative disadvantage in creating and accessing educational opportunities. Burgess et al. [27] have suggested that “stereotype threat” (a process whereby members of a stereotyped group are unconsciously hindered in their performance by awareness of the stereotype) could account for differential attainment by BME students. Woolf et al. [28] have described that BME doctors in the UK can experience poor relationships with seniors and problems fitting in, which can in turn lead to fewer learning opportunities, lower confidence, and an increased chance of mental health problems than experienced by their white counterparts. All of these factors have the potential to reduce performance. As a result, efforts to enhance equality should clarify these mechanisms and design interventions to address them.

It is important not to be unduly reassured by our findings: whilst examiners did not show bias in their scores, feedback or recollections, they did show evidence of stereotype activation. This is consistent with the prior findings of Woolf et al. [11] that medical educators stereotype BME students to some degree. Stereotype *activation* is understood to be a separate cognitive process from stereotype *application*; or more simply, a stereotype can come to mind during a judgement process without influencing the decision that is reached [17]. People are known to be comparatively resistant to stereotype application if they are strongly motivated to avoid prejudice [29], or if they are motivated to achieve accuracy in the task they are performing [30]. It is notable that by not showing an effect of stereotype bias, these findings are at odds with a significant body of research in social psychology. The reasons for this difference are unclear. Differences in study population may offer some explanation. Many social psychology studies have used undergraduates or members of the public, whereas this study recruited qualified doctors. It could be that doctors’ sense of professionalism as examiners or experience of working with trainees from British Asian backgrounds may produce a greater tendency to individuate and thereby resist stereotyping. All such explanations of this difference must, however, be viewed as speculative at this stage, and further work is needed to replicate and understand this difference. At a practical level, it is important to note that whilst these findings help to reassure us that no systematic bias exists in examiners’ judgements, some individual examiners may still exhibit bias (as was indicated by the previously described study by McManus et al. [21]). Equality and diversity training of OSCE examiners has been a mandatory component of assessment in medical education for several years [31]; understanding how such training might have influenced the stereotypes which examiners hold or their motivation to resist applying them to judgements is important to continued efforts to enhance equality.

### Limitations

This study used an adequately powered, double-blinded, randomised, controlled methodology to determine the influence of students’ ethnicity on examiners’ judgements. Consequently, we assert that the study has strong internal validity to address the stated research questions. Despite this, the study has some limitations. The study was (necessarily) conducted in a simulated context, rather than in a real OSCE. It is possible that the pressure of examining in real life could make examiners more vulnerable to stereotyping than they were in this study. The fact that this study is consistent with other observational studies is reassuring in this respect. We compared white students with British Asian students; it is not possible to exclude the possibility that an effect could arise due to other ethnic groups. Examiners in the study only judged three video performances, whereas in real OSCEs examiners may judge a much larger number of performances between breaks. Stereotypes are known to be more influential when individuals’ cognitive resources are depleted [17]; we can’t exclude the possibility that examiners’ judgements could be influenced by students’ ethnicity after a more prolonged series of performances due to (for example) fatigue or lapses in concentration, or that different samples of performance (displaying a different range of behaviours by Asian students) could produce an effect. Lastly, as we did not have a control condition in which participants performed the LDT without watching the videos, we cannot definitively claim that participants activated an Asian stereotype specifically in response to the videos rather than that the activation observed was already present upon beginning the study. These results support the central conclusion that examiners had a stereotype which was active at the time of judging performances, which does not appear to have been applied to their judgements. However, it is possible that stereotype-related differences in judgement are induced only when the behaviours of the individuals being judged contribute directly to further stereotype activation.

### Recommendations for future research

As with all research, this study would benefit from replication by independent groups in other contexts, and using student performances derived from other minority ethnic groups, to determine the generalisability and repeatability of these findings. Further studies with a longer series of performances would help to exclude the possibility of the fatigue-related effect posited above, whilst more investigation is needed of the stereotypes which medical educators appear to possess to understand whether they influence educators’ interactions with BME students in other circumstances. Further research should focus on understanding how BME students’ learning or performance may be disadvantaged within medical education, and whether effective interventions can be developed to ensure equality.

## Conclusions

In this study we have shown that whilst OSCE examiners exhibit evidence of mental stereotypes when examining ethnic minority students, they revealed no evidence that students’ ethnicity (British Asian vs white) has any influence on the scores or feedback that examiners gave to performances. Nor did students’ ethnicity appear to influence examiners’ recollections of performance. Future efforts to address differential attainment by BME students may, therefore, be best directed at understanding detrimental influences on their learning or performance and developing interventions to ensure equality within the learning environment.

## Additional file

Additional file 1:
**Section 1**. Case material and scoring format used in simulated OSCE stations. **Section 2**. Validation results for stimulus materials. (DOCX 20 kb)
