# Supplementary material for: A randomised trial of the influence of racial stereotype bias on examiners’ scores, feedback and recollections in undergraduate clinical exams
Source: BMC Med. 2017 Oct 25;15:179. doi: 10.1186/s12916-017-0943-0 (PMC5655938; doi:10.1186/s12916-017-0943-0)
Supplement: Additional file 1: — Section 1. Case material and scoring format used in simulated OSCE stations. Section 2. Validation results for stimulus materials. (DOCX 20 kb) [file 12916_2017_943_MOESM1_ESM.docx]

Additional file 1

**Section 1: case material and scoring format used in simulated OSCE stations**

**Examiner Information for OSCE station**

Scenario

**This is the information that candidates will read before starting.**

You are currently on an attachment in general practice.

Sally Davies is a 32 year old female patient who has come to the practice today to discuss her recent experiences at the diabetes clinic. Before the consultation starts, you note the following information from the diabetes clinic

“Re: Sally Davies

Ms Davies attended the diabetes clinic today. She describes symptoms of lethargy, polyuria, polydipsia, and weight loss over a 3 month period. Her random blood glucose was 22 in primary care, and was 18 again today. Her urine showed only a trace of ketones. She has been diagnosed with type 1 diabetes. She has received education by our specialist nurses, and started on a combination of long acting once daily insulin and short acting insulin with meals. We’ll review her again shortly.

Yours sincerely”

**Please have a discussion with Sally about her illness and address her concerns.**

**Information for Standardised Patient:**

You are a 32 year woman. Up until recently you have always kept in good health. You had the usual coughs and colds as a child, but were never seriously unwell.

About 3 months ago you noticed that you had started waking up in the night to pass urine, which is not something that you have often done before. You also noticed yourself feeling thirsty a lot, and drinking a lot of water and soft drinks. This seemed to explain why you had to go to the toilet so often. As time went by, you also started to feel tired a lot; you just lacked your usual energy, and things were becoming a real effort. You also noted that you had dropped a dress size, and whilst you weren’t displeased by this, you thought it was odd, as you’ve always found losing weight difficult.

A week ago you went to see your GP, and they thought you might have diabetes. They tested you urine, which they said had a lot of sugar in it and then they sent you to the diabetes clinic at the hospital, where they have started you on insulin injections. You’ve been doing these every day since, but you really don’t like them. They hurt, and you feel embarrassed doing them at work. At least you are feeling a bit better now, and less thirsty and tired!

*Past Medical History:* Nothing

*Medication:* You take the oral contraceptive pill

*Alllergies to medication:* None

*Social history:* Married, no children. Your husband is well.

You smoke 10-15 cigarettes / day. You smoke cannabis a few times a month, although you used to smoke more of it in your early 20s. You work as an interior designer, which involves a mix of detailed work at a computer, and a lot of driving to locations. You couldn’t do your work without a car. You drink 15-20 units of alcohol per week – mostly red wine. You rarely take exercise.

*Ideas concerns and expectations:*

You have a number of concerns. You feel overwhelmed and worried by all of this. In particular you have some queries: Firstly, they gave you rules about what to do if you’re ill. They told you to **increase** your insulin if you’re vomiting, which seemed like the wrong advice. You think if you’re not eating you should probably miss the insulin out. Secondly, you’ve looked on the internet and seen that people with diabetes can go blind. You’re really scared about this, particularly as your work requires detailed visual focus. Mostly you just want the doctor to listen, and to explain clearly about your new diagnosis.

**Scoring Format:**

**Please use this scale to allocates scores to each student’s performance**

**Please note:** intermediate marks may be awarded for either 1/. missing elements or 2/. because some elements were not done well.

**Approach to communication:**

| No elements done | | |  |  |  | All elements done well | | |
| --- | --- | --- | --- | --- | --- | --- | --- | --- |
|  | 1 | 2 | 3 | 4 | 5 | 6 | 7 |  |

Clarifies patient’s current understanding

Establishes what the patient would like to address

Delivers information in appropriate language

Gives information in step-wise manner

Displays interest and attention

Encourages patient to speak (verbal prompts / nodding / avoiding interruption)

**Accuracy of explanation of condition:**

| No elements done | | |  |  |  | All elements done well | | |
| --- | --- | --- | --- | --- | --- | --- | --- | --- |
|  | 1 | 2 | 3 | 4 | 5 | 6 | 7 |  |

Diabetes is an autoimmune condition

Pancreatic destruction produces a complete lack of insulin

Insulin maintains blood glucose by enabling glucose to enter cells.

Lack of insulin can make a person very unwell.

Lifelong treatment with insulin is necessary

Complications arise from poor long term glucose control

**Response to patient’s emotions and concerns:**

| No elements done | | |  |  |  | All elements done well | | |
| --- | --- | --- | --- | --- | --- | --- | --- | --- |
|  | 1 | 2 | 3 | 4 | 5 | 6 | 7 |  |

Elicits and responds to patients concerns

Avoids dismissing or minimising concerns

Avoids false or premature reassurance

Recognises patients emotional responses

Demonstrates understanding and empathy

Displays sensitivity and tact.

**Factual answers to patients concerns:**

| No elements done | | |  |  |  | All elements done well | | |
| --- | --- | --- | --- | --- | --- | --- | --- | --- |
|  | 1 | 2 | 3 | 4 | 5 | 6 | 7 |  |

Complications can involve: eyes / kidneys / nerves / heart attacks / strokes / PVD

Diabetic retinopathy is common by 10-20 yrs

Good glycaemic control can help to avoid retinopathy

Laser therapy can also be used to treat some diabetic eye complications

Insulin should be **increased** when a person with diabetes is unwell

**Overall Performance:**

| Fail | | Borderline | Pass | Good | Excellent | |
| --- | --- | --- | --- | --- | --- | --- |
| 1 | 2 | 3 | 4 | 5 | 6 | 7 |

**Section 2: validation results for stimulus materials**

There were three separate stimuli within the methods that required validation: the constructs within the scripts and the degree to which the videos of Asian and white students’ performances were similar; the degree to which the words used in the lexical decision task did indeed evoke the intended constructs of “Asian” or “neutral”; and the degree to which the phrases in the recollection task represented either good communication, poor communication, accurate factual knowledge or inaccurate factual knowledge.

**Validation of scripted constructs and similarity of video performances:**

Review of the scripts was performed by a panel of 6 experienced clinical educators. All 6 had extensive experience of OSCE examining. All 6 indicated that they supported the authenticity of the scripts. The mixed script was edited based on initial ratings, as it portrayed a standard of better which was better than intended. Following revision, the ratings of the final scripts were as follows:

| Script | Scores given to each script by domain  Mean (Min-Max) | | |
| --- | --- | --- | --- |
|  | Communication | Knowledge | Overall |
| K+/C- | 2.3 (2.0-3.0) | 5.5 (4.5-6.5) | 2.8 (2.0-4.0) |
| Mixed | 3.4 (2.0-5.5) | 3.9 (3.5-5.0) | 3.1 (2.0-4.0) |
| K-/C+ | 6.2 (4.5-7.0) | 3.8 (2.5-5.0) | 4.2 (2.0-6.0) |

On this basis the scripts were judge to represent the intended constructs.

A panel of 8 clinical educators was recruited to review the videos. The panel comprised 5 individuals of white ethnicity and 3 individuals of Asian ethnicity. The group also comprised 4 men and 4 women and ranged in clinical seniority from speciality trainee level to senior consultants / GPs. Review panel members were asked to watch each pair of videos, deliberately comparing the communication and factual knowledge which they demonstrated, and then separately rate the similarity of the communication and factual knowledge on the following scale: Completely Identical (5), Nearly Identical (4), Highly Similar (3), Fairly Similar (2), Little Similarity (1). Each participant scored the similarity of each pair of videos before moving to the next pair of videos. The presentation order of videos within pairs and the order of the pairs themselves were counter-balanced between different participants. The following median ratings and ranges of video similarity were obtained:

|  | Median Rating of Similarity of Communication  (range) | Median Rating of Similarity of Knowledge  (range) |
| --- | --- | --- |
| Performance 1  (K+/C-) | 3.5  (3-5) | 5  (4-5) |
| Performance 2  (K-/C+) | 4  (3-5) | 5  (4-5) |
| Performance 3  (Mixed) | 4  (2-5) | 5  (4-5) |

Consequently all pairs were rated highly similar or better on both dimensions. On this basis the similarity of the videos was judged to be sufficient to use in the study.

**Validation of Asian and neutral constructs for lexical decision task:**

A list of 28 candidate “Asian” words and 28 candidate “neutral” words were selected from a mixture of academic literature, social media and internet forums which described the content of stereotypes of Asian people in the UK. The same panel of clinician educators was asked to rate each word depending on how strongly it was associated with either their own or popular conceptions / stereotypes of “Asian-ness” in the UK, on a 5-point end-anchored Likert scale that ranged from 1 – not at all associated with Asian-ness to 5 – highly associated with Asian-ness. The words were presented to all reviewers as the same, randomly ordered list. 15 words in each category were selected to give “Neutral” words with the lowest scores and “Asian” words with the highest scores, whilst ensuring that all words were matched for letter length in in the corresponding category. The resulting lists were, “Asian” words: spices, multicultural, Delhi, curry, Sari, mosque, cricket, traditional, immigration, family, Lahore, newsagent, turban, respectful, cultural; “neutral” words: badger, anachronistic, Paris, token, sofa, office, linkage, objectively, synthesizer, embryo, Naples, lubricant, carpet, glistening, dramatic. Their corresponding median ratings of “Asian-ness” (with low and high inter-quartile ranges) were: “Asian” words, 3.5 (3.0-4.0); and “neutral” words, 1.0 (1.0-1.0), p=0.001 (Fishers exact). On this basis the constructs were judged to adequately represent the constructs to be used in the study. The non-words that were selected were: chorts, phrict, swund, phlonned, japhed, nolfs, gwitchedsneap, gwoe, bledged, iscaisnet, shraffeding, prorld, glourswhape, suised, zirstronly.

**Validation of good and poor communication and knowledge statements for test of recollection**

One review panel member dropped out for the validation of the recollection phrases, leaving 7 of the original panel members. These reviewers were asked to judge the 40 quotes used in the test of recollection. 20 of these statements were real excerpts from the script of the “mixed” performance, and 20 were spurious but plausible inventions which did not appear in any performance. The statements were presented to all reviewers as the same, randomly ordered list. For each statement, the reviewers were asked to make a categorical judgement as to whether the statement represented “good communication”, “poor communication”, “good factual knowledge”, or “poor factual knowledge”. Reviewers were supplied with the same examiner information sheet as study participants, to assist in judgements of good or poor factual knowledge in the context of the simulated OSCE station used in the study. Categorical responses were collated from all 7 reviewers and compared with the intended constructs. Median agreements with each construct were: real, stereotype consistent phrases 93%; real, stereotype inconsistent phrases 86%; invented, stereotype consistent phrases 93%; invented, stereotype inconsistent phrases 100%. Individual Cohen’s Kappa agreement was calculated between each reviewer’s responses and the intended construct, and then a weighted average of these individual Cohen’s Kappa’s was calculated. Individual values of Kappa ranged from 0.67 to 0.90, with a weight average of 0.71. On this basis, these phrases were judged to adequately represent the intended construct to use in the study.
